# Vaginal Foreign Body Insertion in Adolescents Influenced by Social Media: A Three‐Case Report

**DOI:** 10.1002/ccr3.72360

**Published:** 2026-04-23

**Authors:** Fatemeh Keikha, Huma Homam, Hawraa Hassan Shbeeb, Rana Karimi, Azadeh Tarafdari

**Affiliations:** ^1^ Department of Obstetrics and Gynecology, Imam Khomeini Hospital Complex, School of Medicine Tehran University of Medical Sciences Tehran Iran; ^2^ Department of Obstetrics and Gynecology, Arash Women's Hospital, School of Medicine Tehran University of Medical Sciences Tehran Iran

**Keywords:** adolescents, foreign body insertion, psychosocial impacts, social media, vagina

## Abstract

Foreign body insertion into the vagina among adolescents, though uncommon, presents unique diagnostic and management challenges. In younger children, diagnosis is often challenging because they may be unable to recall or report foreign body insertion. In contrast, adolescents more frequently acknowledge insertion, making diagnosis comparatively less complex. Here, we discuss three cases of adolescents who inserted objects under the influence of social media content or emotional distress. Each patient reported persistent, nonspecific symptoms that led to delayed diagnosis. Ultrasound imaging and vaginoscopic evaluation under anesthesia were ultimately necessary for definitive identification and removal. Psychosocial assessment revealed underlying contributors such as sexual curiosity, low mood, and social media‐driven behaviors. These cases underscore the importance of a high index of suspicion for unexplained genitourinary complaints, prompt multidimensional evaluation including vaginoscopy, and a comprehensive team approach incorporating gynecological, pediatrics, psychological, and family support. In the era of evolving digital media influences, clinicians should remain vigilant about psychosocial factors contributing to adolescent risk‐taking behavior.

## Key Clinical Message

When adolescents voluntarily disclose vaginal foreign body insertion, diagnosis is facilitated and timely management can be achieved. Even when imaging findings are conclusive, vaginoscopy remains essential to ensure complete removal and assess for potential vaginal mucosal injury. Integrated psychological support and proactive family involvement are crucial to prevent recurrence.

## 1 Introduction

Although vaginal foreign body insertion can occur at any age, including infancy, the majority of reported cases involve adolescents and young adults due to factors such as curiosity, impulsivity, or social media influences [1, 2]. Vaginal foreign body insertion represents a rare but clinically significant condition, with reported cases predominantly affecting pediatric and adolescent populations. Diagnosis is often delayed because of nonspecific symptoms, such as vaginal discharge or bleeding, and may result in complications including infection, mucosal injury, or psychological distress. Prompt recognition, appropriate imaging, and vaginoscopic evaluation are critical for effective management and favorable outcomes. The following three cases describe adolescents in their early teens who inserted various objects, leading to complications such as infection, psychological distress, and social stigma. These reports highlight the importance of timely clinical intervention, comprehensive psychological evaluation, and sustained follow‐up to address underlying triggers and prevent recurrence.

## 2 Case Presentations

### 2.1 Case 1

A 13‐year‐old single, virgin girl, followed by her parents, arrived at the emergency department with a three‐month history of malodorous yellow vaginal discharge. During the three‐month symptom duration, no prior medical evaluation or treatment was sought before presentation to our center, as the patient initially refrained from disclosing the incident to her mother because of embarrassment and fear. She admitted to inserting a bead into her vagina but was unable to remove it. She reported regular menstrual cycles since menarche at age 11, with no pain, itching, or fever. Her past medical and psychological history was unremarkable.

On physical examination, there was mild erythema in the posterior fourchette and yellow discharge. Because her hymen was intact, speculum and bimanual examinations were deferred. Laboratory tests (complete blood count and urinalysis) were normal. The patient was admitted for diagnostic vaginoscopy, and evaluation by a pediatrician and a psychologist.

The pediatric examination was unremarkable. The psychologist ruled out ADHD (Attention‐Deficit/Hyperactivity Disorder), mood disorders, anxiety disorders, and other psychiatric conditions. During the interview, the patient disclosed that she felt sexual arousal when viewing explicit social media content and inserted objects to relieve this sensation.

**FIGURE 1:**
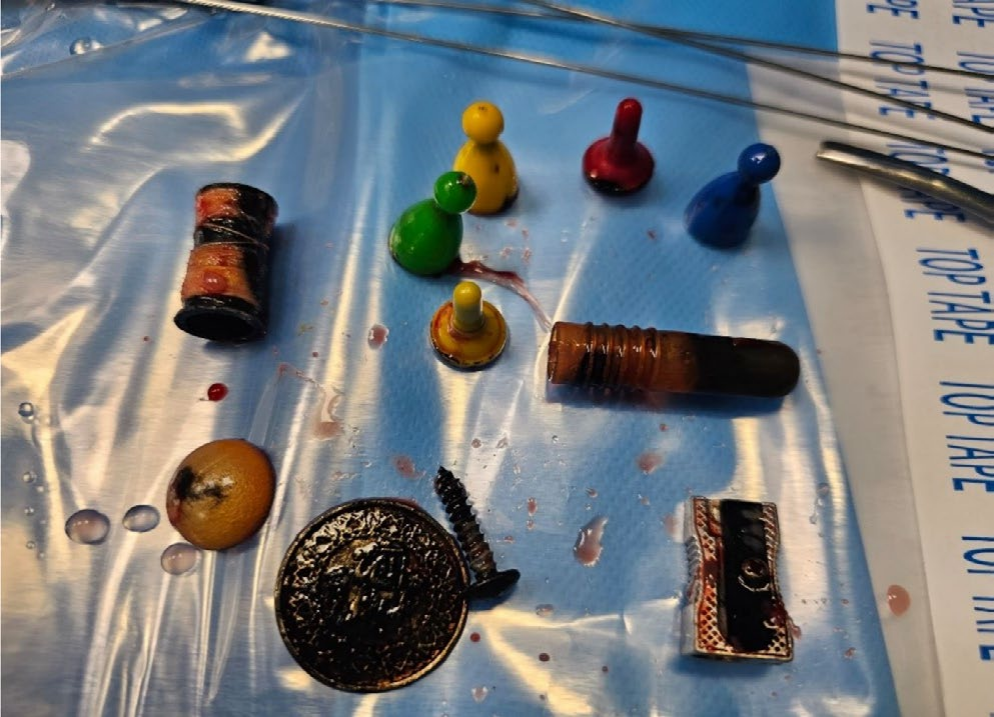
Showing multiple foreign bodies meticulously extracted from the vagina of the virgin girl.

Under anesthesia, vaginoscopy revealed multiple foreign objects, including small game pawns in different colors (yellow, green, red, and blue), a wooden spool, a metallic screw, a coin, a button, a sharpener, and another slender metallic piece (Figure 1). We discovered a single bead at the external cervical orifice. All objects were carefully removed under direct visualization. Hysteroscopy confirmed a normal uterine cavity and visible tubal ostia.

The gynecology and psychology teams counseled the patient and her parents on the complications of foreign body retention, including infection, trauma, and possible infertility. Safe sexual practices and coping strategies for managing arousal were discussed. The patient reported deleting triggering social media applications from her phone.

A comprehensive follow‐up plan was created, including regular medical check‐ups, therapy sessions to address emotional triggers, and family support. Although follow‐up was recommended and discussed with the parents, the patient was subsequently lost to follow‐up, which may reflect parental reluctance or non‐compliance with ongoing management. Attempts to contact her were unsuccessful, and her parents expressed reluctance to continue care due to stigma. They ceased communication with the medical team, raising concerns about her mental health and the risk of recurring behavior without proper intervention.

### 2.2 Case 2

A 13‐year‐old single, non‐virgin girl presented to the emergency department after inserting a jam jar into her vagina the previous day. She reported no pain, vaginal discharge, or bleeding. Her last menstrual period occurred 12 days prior, and she had regular 30‐day cycles with a seven‐day flow. Her past medical history was unremarkable, except for abnormal uterine bleeding managed with medroxyprogesterone 10 mg daily for 14 days each month. Although she mentioned low mood, she had no acute psychiatric complaints on admission.

**FIGURE 2:**
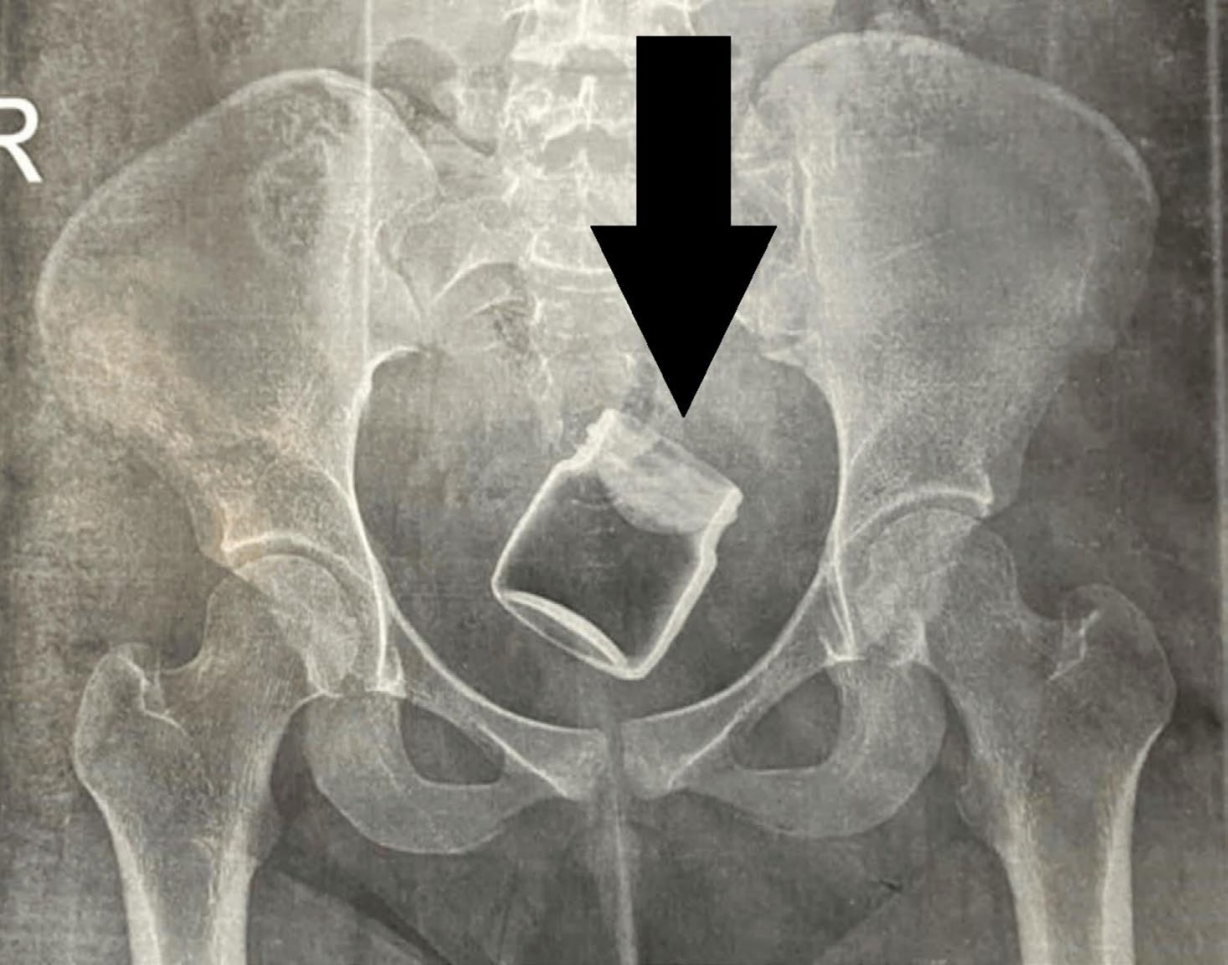
Plain radiograph revealing a well‐defined, radiopaque object consistent with a jam jar situated in the mid‐pelvic cavity.

On examination, her vital signs were within normal limits. She was obese but had no other significant findings on head, neck, chest, or abdominal evaluations. Perineal inspection revealed no visible injuries or discharge. A pelvic ultrasound showed a normal uterus, cervix, and adnexa, along with a circular echogenic object between the vaginal introitus and the cervix. An X‐ray confirmed the presence of a jam jar measuring approximately 6 cm × 6 cm in the mid‐pelvis (Figure 2).

The patient was admitted for surgical removal of the foreign body, and a psychological consultation was requested. According to the psychologist's assessment, she had a history of childhood hyperactivity, attention deficits, and periodic depressive episodes. She frequently masturbated using various objects, often stimulated by images and videos on social media. She described ongoing irritability, anxiety, and prolonged low mood. Before this incident, she was angered by her parents' refusal to meet her demands, locked herself in her room, and inserted the jam jar in response to her frustration. The psychologist prescribed fluoxetine and olanzapine, scheduling a follow‐up in two weeks.

In the operating room, the patient underwent general anesthesia in the lithotomy position. Examination revealed an old hymenal laceration at the 6 o'clock position but no fresh tears or abnormal discharge. The jar was found upside down and proved difficult to extract. By applying combined rectal and vaginal pressure, the surgical team successfully removed it. Subsequent hysteroscopic evaluation showed no injuries to the vagina or cervix; the endometrium was normal, and both tubal ostia were visible.

The patient recovered without complications and was discharged the following day with instructions to return for a one‐week follow‐up appointment. However, despite repeated attempts to contact her, neither she nor her family responded, likely due to embarrassment and social stigma.

### 2.3 Case 3

An 11‐year‐old single non‐virgin girl presented to the day clinic one day after inserting a U‐shaped hairpin into her vagina. She reported no pain, vaginal discharge, or bleeding. Menarche occurred two years ago, and she had regular 30‐day cycles with a seven‐day flow. She had no significant past medical history and no acute psychiatric complaints at admission.

**FIGURE 3:**
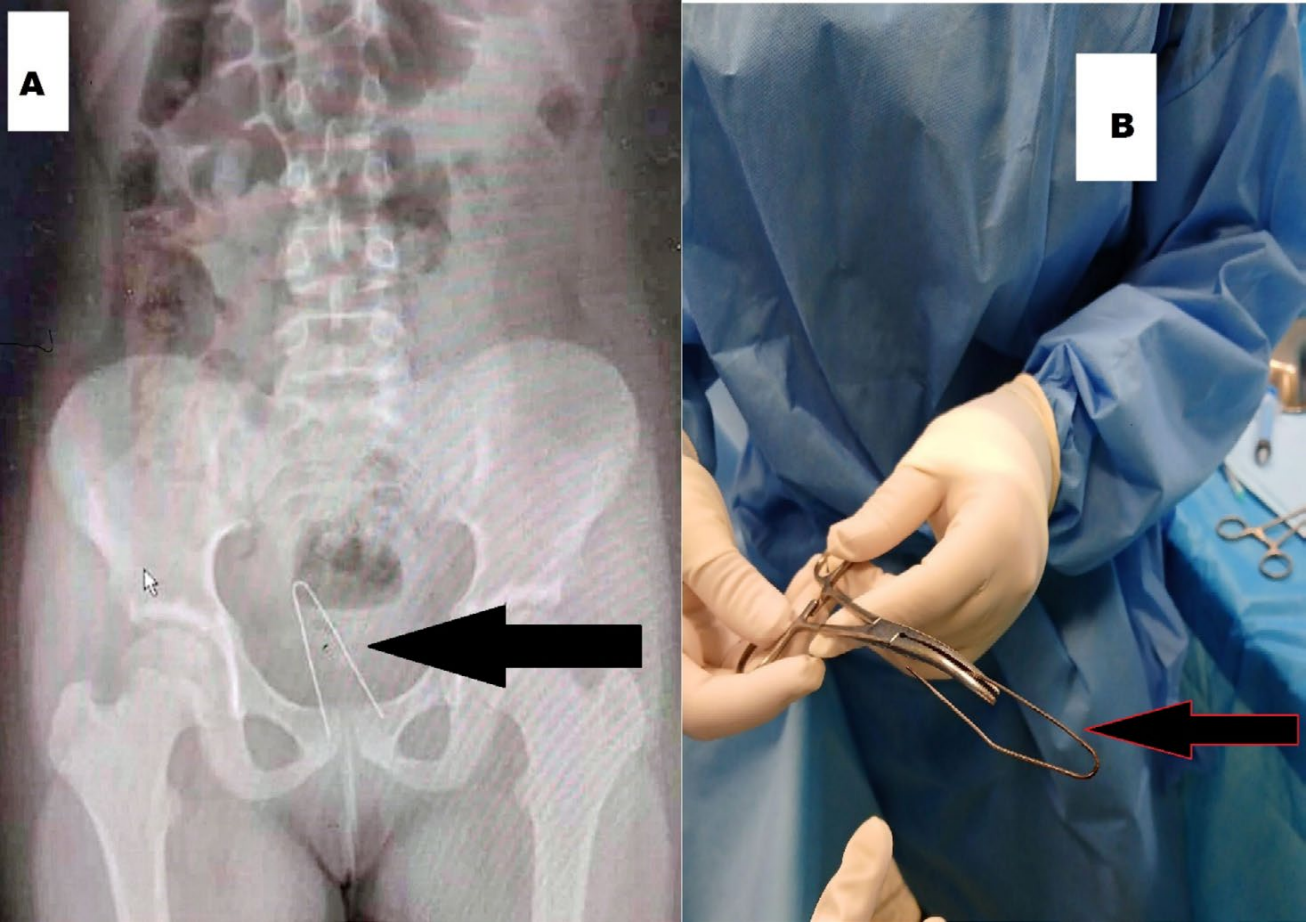
(A) Radiographic image demonstrating the presence of a U‐shaped hairpin located in the patient's mid‐pelvic region. (B) Showing the U‐shaped hairpin that was successfully extracted by the surgical team.

On examination, her vital signs were within normal limits. She appeared thin and somewhat short for her age but otherwise healthy. Head, neck, chest, and abdominal exams were unremarkable. Perineal inspection showed no visible injuries or discharge. A transabdominal ultrasound revealed a normal uterus, cervix, and adnexa with no foreign objects. However, an X‐ray confirmed the presence of a U‐shaped hairpin in the mid‐pelvis (Figure 3A).

She was admitted for surgical removal of the foreign body, and a psychological consultation was obtained. According to the psychologist, she had a history of childhood hyperactivity but showed no signs of depression or other psychiatric disorders. She mentioned occasional aggressive behavior toward her older sister and admitted to locking herself in her room for hours. She denied any previous history of inserting objects into her vagina but said she would sometimes explore her vulva and vagina without realizing it could harm her hymen. She also denied any form of abuse or sexual experience.

In the operating room, the patient was placed under general anesthesia in the lithotomy position. Examination revealed an old hymenal laceration at the 11 and 2 o'clock positions, but no fresh tears or abnormal discharge. Under hysteroscopic guidance, the hairpin was found lodged in the vaginal wall. Although removal was initially difficult, the surgical team successfully extracted it (Figure 3B). A thorough hysteroscopic exam confirmed no injuries to the vagina or cervix; the endometrium appeared normal, and both tubal ostia were visible.

She recovered without complications and was discharged the following day with instructions to return for a follow‐up appointment in two weeks. At her follow‐up visit, she was in good spirits and stated she would not repeat the behavior. Since she reported no further issues, both the obstetrics/gynecology team and the psychiatry team concluded her follow‐up for this incident.

## 3 Discussion

Vaginal foreign body (VFB) in adolescents is increasingly recognized as a complex clinical issue that intersects gynecologic, psychosocial, and behavioral factors. Several recent systematic reviews underscore that VFBs pose unique diagnostic and management challenges, often presenting with nonspecific symptoms like discharge, bleeding, or persistent genitourinary complaints [1]. In the current cases, the insertion of foreign objects was associated with curiosity, anger, or sexual arousal while viewing explicit content on social media, echoing observations that social media can reinforce impulsive behavior, encourage experimentation, and, in some instances, normalize risky practices [2].

One recurring challenge in VFB cases is missed or delayed diagnosis. In contrast, a notable feature of the present case series was that all three patients clearly acknowledged vaginal foreign body insertion, which facilitated prompt diagnosis and expedited management. Advanced imaging such as magnetic resonance imaging (MRI) or ultrasonography can aid evaluation but may fail to detect small or concealed objects [3]. As shown in our cases, the definitive discovery often hinges on vaginoscopy or a careful manual exam under anesthesia, underscoring calls to prioritize thorough pelvic assessments—particularly when the history suggests persistent vulvovaginal symptoms despite negative imaging [1, 3].

Vaginoscopy plays a pivotal role in both the diagnosis and management of vaginal foreign bodies in pediatric and adolescent patients. It enables direct visualization of the vaginal cavity, facilitates safe and complete removal of foreign objects, and allows thorough assessment of the vaginal mucosa for associated injuries or complications. As previously described, vaginoscopy offers superior diagnostic accuracy compared with imaging alone and is particularly valuable when clinical suspicion persists despite inconclusive radiologic findings. Furthermore, its minimally invasive nature and effectiveness make it the preferred approach for evaluation and management in this population [4].

Although many VFB cases arise from exploration or accidental insertion, recent data highlight that social media exposure can play a key part in shaping sexual behavior and curiosity among youth [2]. This exposure can be beneficial in providing online support communities and helpful content, but it can also expose adolescents to viral “challenges,” negative peer pressure, or explicit sexual material. Studies indicate that adolescents often lack the maturity to filter harmful content effectively, making them more prone to impulsive acts like foreign body insertion [5, 6]. Furthermore, unmonitored social media use may contribute to low self‐esteem and self‐harm risk, both of which can manifest in abnormal or risky sexual behaviors [2].

Experts consistently emphasize a team‐based strategy, involving gynecologists, pediatricians, psychologists, and sometimes child protective services [5]. Such coordination ensures a thorough medical and psychosocial evaluation, recognizing that some adolescents might have underlying emotional or psychiatric needs. In our cases, psychological input was crucial both for assessing possible mood disorders and for guiding long‐term support. This aligns with findings that adolescents require tailored interventions that address not only the immediate physical removal of the object but also the circumstances—such as social media practices or family conflicts—that prompted insertion [1, 6].

Recent literature highlights the importance of proactive parental involvement and supervised online activity in reducing the risk of vaginal foreign body and other harmful behaviors [2]. Engaging families in discussions about digital media limitations, encouraging healthy coping strategies, and promoting open, stigma‐free dialogues around sexual development can help mitigate unsafe experimentation. Active parental engagement is also essential to ensure adherence to follow‐up care, reduce stigma‐related barriers to treatment, and support long‐term behavioral prevention strategies. In line with published recommendations, our multidisciplinary teams emphasized education on safe sexual practices, mental health referrals, and family‐based counseling for these patients [5, 6].

## 4 Conclusion

Altogether, our three cases underscore the multifaceted challenges in diagnosing and managing VFB in adolescents, particularly when social media influences and emotional distress are present. A high index of suspicion is warranted for recurrent or unexplained genitourinary complaints, and definitive evaluation with vaginoscopy under anesthesia can be critical [3]. More broadly, clinicians should address the psychosocial context—including social media consumption—that may propel adolescents toward high‐risk behaviors, consistent with emerging best practices in the literature [1, 2]. By integrating medical, psychological, and educational efforts, we can more effectively promote safety, timely diagnosis, and holistic care for adolescent patients faced with these complex issues.

## Author Contributions

**Fatemeh Keikha:** conceptualization, investigation, project administration, supervision, validation, writing – review and editing. **Huma Homam:** conceptualization, data curation, investigation, project administration, resources, supervision, validation, writing – original draft, writing – review and editing. **Hawraa Hassan Shbeeb:** conceptualization, data curation, investigation, project administration, resources, supervision, validation, writing – original draft, writing – review and editing. **Rana Karimi:** investigation, resources, writing – original draft. **Azadeh Tarafdari:** conceptualization, data curation, investigation, project administration, resources, supervision, validation, writing – review and editing.

## Funding

This work received no specific grant from any funding agency. All efforts were part of routine clinical practice and academic endeavor by the authors.

## Ethics Statement

We strictly adhered to the principles of the Declaration of Helsinki throughout the whole study process. Also, this study was approved by the Research and Ethics Committee of the Tehran University of Medical Sciences.

## Consent

Written and formal consent for the publication of this case report was obtained from the patient.

## Conflicts of Interest

The authors declare no conflicts of interest.

## Data Availability

Data sharing is not applicable to this article as no datasets were developed or analyzed during the study.
